# Modulation of NKp30- and NKp46-Mediated Natural Killer Cell Responses by Poxviral Hemagglutinin

**DOI:** 10.1371/journal.ppat.1002195

**Published:** 2011-08-25

**Authors:** Mostafa Jarahian, Manuela Fiedler, André Cohnen, Dominik Djandji, Günter J. Hämmerling, Cornelius Gati, Adelheid Cerwenka, Peter C. Turner, Richard W. Moyer, Carsten Watzl, Hartmut Hengel, Frank Momburg

**Affiliations:** 1 Translational Immunology Research Unit, German Cancer Research Center, Heidelberg, Germany; 2 Institute for Virology, Heinrich-Heine-University, Düsseldorf, Germany; 3 Institute of Immunology, University of Heidelberg, Heidelberg, Germany; 4 Department of Molecular Immunology, German Cancer Research Center, Heidelberg, Germany; 5 Innate Immunity Junior Research Group, German Cancer Research Center, Heidelberg, Germany; 6 Department of Molecular Genetics and Microbiology, University of Florida College of Medicine, Gainesville, Florida, United States of America; Washington University School of Medicine, United States of America

## Abstract

Natural killer (NK) cells are an important element in the immune defense against the orthopox family members vaccinia virus (VV) and ectromelia virus (ECTV). NK cells are regulated through inhibitory and activating signaling receptors, the latter involving NKG2D and the natural cytotoxicity receptors (NCR), NKp46, NKp44 and NKp30. Here we report that VV infection results in an upregulation of ligand structures for NKp30 and NKp46 on infected cells, whereas the binding of NKp44 and NKG2D was not significantly affected. Likewise, infection with ectromelia virus (ECTV), the mousepox agent, enhanced binding of NKp30 and, to a lesser extent, NKp46. The hemagglutinin (HA) molecules from VV and ECTV, which are known virulence factors, were identified as novel ligands for NKp30 and NKp46. Using NK cells with selectively silenced NCR expression and NCR-CD3ζ reporter cells, we observed that HA present on the surface of VV-infected cells, or in the form of recombinant soluble protein, was able to block NKp30-triggered activation, whereas it stimulated the activation through NKp46. The net effect of this complex influence on NK cell activity resulted in a decreased NK lysis susceptibility of infected cells at late time points of VV infection when HA was expression was pronounced. We conclude that poxviral HA represents a conserved ligand of NCR, exerting a novel immune escape mechanism through its blocking effect on NKp30-mediated activation at a late stage of infection.

## Author Summary

Natural killer (NK) cells, which belong to the innate immune system, play a critical role in the defence against viruses. The orthopoxvirus family member vaccinia virus is not only used for vaccinations of humans, but is also presently considered as a promising agent for oncolytic virotherapy of tumors. It is, therefore, of importance to better understand mechanisms involved in recognition and lysis of vaccinia virus-infected cells by NK cells. In this study, we have identified the hemagglutinin molecule from vaccinia virus and the related mousepox virus as a novel interaction partner for two activating receptor molecules on NK cells. As one of these receptors was blocked by hemagglutinin, vaccinia-infected cells were less effectively killed by human NK cells than uninfected cells. Our findings support the use of hemagglutinin-deficient VV variants for oncolytic therapy.

## Introduction

Vaccinia virus (VV) is an extensively studied, prototypic member of the *Poxviridae* family. It is a large virus with a double-stranded DNA genome of ∼200 kbp encoding ∼250 genes [1]. VV has a broad cellular tropism and infects almost any cell line in culture [1]. VV is highly immunogenic and has been successfully used to vaccinate against smallpox [2]. Vaccinia-derived vectors have also been extensively used as expression vectors for foreign genes and as recombinant vaccines [3]. In spite of various immune evasion mechanisms [4], [5], VV and other poxviruses elicit strong humoral and cellular immune responses [6]–[10].

Natural killer (NK) cells play an important role in protective immune responses against VV [6], [11], [12] and the ectromelia mousepox virus (ECTV) [13], [14]. Interferon(IFN)-γ secretion by NK and non-NK cells appears to be involved in the antiviral effect [6], [14], [15]. Type I interferons are essential for the activation of NK cells against VV [16], [17]. Recently, it has been reported that VV infection induces ligands for the activating natural cytotoxicity receptors (NCR), NKp46, NKp44 and NKp30, and increases susceptibility to lysis by NK cells [18]. VV-induced NCR ligand(s) were described to appear early during infection but have not been identified on a molecular level. Furthermore, it was shown that the activating NK cell receptor NKG2D is involved in the NK-cell mediated resistance to poxvirus disease in C57BL/6 mice [19]. Expression of NKG2D ligands was reported to be enhanced by ECTV infection [19].

The functions of NK cells are regulated through a balance of activating and inhibitory signals, which are transmitted through particular receptors binding cytokines or ligand structures on interacting target cells and pathogens [20], [21]. Most inhibitory receptors recognize particular MHC class I isoforms and thereby ensure tolerance of NK cells against self antigens [22]. CD16, NKG2D, the natural cytotoxicity receptors (NCR) NKp30, NKp44 and NKp46, as well as NKp80, DNAM-1, and various costimulatory receptors are involved in the activation of human NK cells [20], [21].

NCR are important activating receptors for the anti-tumor and anti-viral activity of NK cells [20], [21], [23]. Heparan sulfate proteoglycans have been described as ligand structures for NKp46, NKp44 and NKp30 [24]–[26]. Nuclear factor BAT3, which is released from tumor cells under stress conditions, and a member of the B7 family, B7-H6, have been identified as cellular ligands for NKp30 [27], [28]. We reported that ligands for NKp30 and NKp44 can be detected on the surface and in intracellular compartments of several kinds of tumor cells [29]. Several NCR ligands derived from pathogens have been described. The hemagglutinin protein of influenza and the hemagglutinin-neuraminidase of Sendai virus and Newcastle disease virus can bind to NKp46 and NKp44 and activate NK cells [30]–[33]. The pp65 matrix protein of human cytomegalovirus (HCMV) has been shown to bind NKp30 and inhibit its function [34]. In addition to VV, human immunodeficiency virus and herpes simplex virus have also been demonstrated to upregulate the expression of NCR ligands in infected cells [35], [36].

Attenuated VV strains are employed to specifically infect and destroy carcinoma cells in xenograft mouse models [37], [38]. Depending on the route of administration VV elicits strong immune reactions at the site of infection involving γδ-T cells, macrophages and NK cells [8], [11], [17], [39]. The predominance of particular cytokines and chemokines acting on NK cells suggests that tumor regression may, in part, be induced by NK cell cytotoxicity in cooperation with the viral oncolytic process [40], [41]. Therefore, it seemed important to study the direct interaction of poxvirus-infected tumor cells with NK cells and elucidate molecular mechanisms involved in this interaction.

Here we have identified the HA molecules from VV and ECTV as novel viral ligand structures for NKp30 and NKp46. While NK cell activation through NKp30 was found to be blocked by HA, NKp46 was triggered by HA. VV-infected tumor cells showed a decreased lysis susceptibility, suggesting that NK cell inhibition through HA was a dominant factor.

## Results

### Poxviral infection induces ligands for NKp30 and NKp46

**Figure 1:**
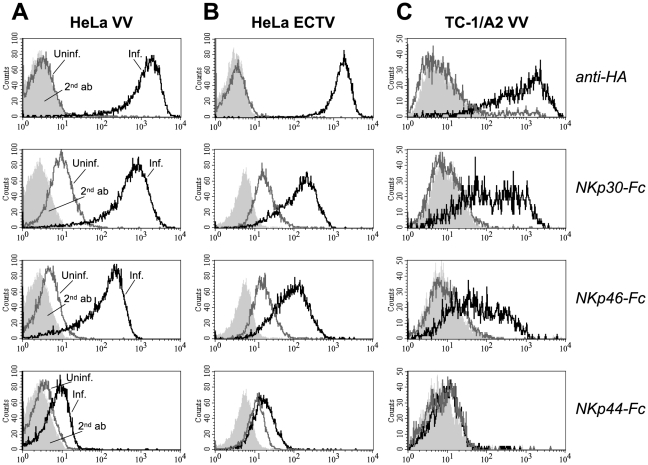
Infection with vaccinia (VV) and ectromelia virus (ECTV) induces ligand structures for NKp30 and NKp46. Human HeLa cervix carcinoma (A, B) and mouse TC-1 lung carcinoma cells (C) were infected with VV strain WR (A, C) and ECTV strain MP-Nü (B) for 20 h, or left uninfected. *Top row*, infected (*black lines*) and uninfected cells (*gray lines*) were labeled with the anti-hemagglutinin (HA) mAb VVI-4G9 to control for the efficiency of infection, or goat anti-mouse Ig-PE secondary antibodies only (*light gray, filled*) and analyzed by cytofluorometry. *2nd to 4th row*, Infected and uninfected cells were stained as indicated with soluble chimeric receptors consisting of the ectodomains of NKp30, NKp46 or NKp44, and the Fc portion of human IgG1. Stainings with goat anti-mouse or anti-human IgG-PE secondary antibody controls are shown as filled histograms.

Using soluble NCR ectodomains fused to the Fc portion of human IgG1 [33] for immunofluorescence stainings, we observed that upon infection of the human cancer line HeLa with the poxviruses, vaccinia virus (VV) or ectromelia virus (ECTV), surface expression of ligand structures for the NK receptors NKp30 and NKp46 was strongly induced as compared with uninfected cells (Figure 1A,B). The efficiency of infection was assessed using the monoclonal antibody (mAb) VVI-4G9 to VV HA, which cross-reacted with the closely related, ECTV-derived HA molecule. NKp30 and NKp46 ligands were more markedly induced by VV than by ECTV infection. Poxviral infection, however, only weakly enhanced the surface expression of ligands for NKp44. Similar results were obtained when infecting other human carcinoma cell lines such as A549 and PANC-1 or melanoma lines with VV or ECTV (see below and data not shown). We also confirmed previous findings [18], that VV infection induced NKp30 and NKp46 ligand expression in MRC-5 human fetal lung fibroblasts as well as in primary, human foreskin fibroblasts (Figure S1), noted, however, that the permissivity to VV infection as judged by HA expression, and the upregulation of NKp30/NKp46 ligands was much lower than in HeLa cells used in the same experiment. The murine lung tumor line TC-1 was labeled by NKp30-Fc and NKp46-Fc after VV infection (Figure 1C), too, suggesting that involved ligand structures were of viral origin rather than being human host cell-derived proteins.

### HA-deficient VV fails to induce NCR ligands

**Figure 2:**
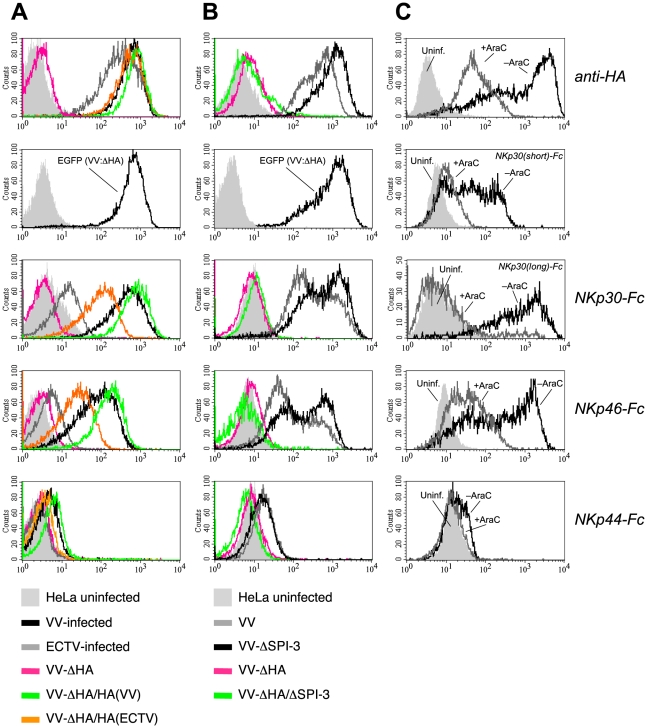
Increased binding of NKp30-Fc and NKp46-Fc is dependent on HA expression. (A) HeLa cells were infected for 18 h with wild-type VV(WR) (*black*), ECTV (*gray*), a HA-deletion mutant of VV (*pink*) as well as with this ΔHA mutant reconstituted with the HA genes from VV(WR) (*green*) and ECTV (*orange*). Stainings of uninfected cells are shown as filled gray histograms. Infection efficiencies were monitored by staining against the late-phase viral product HA (*top row*) or EGFP (*second row*), which was inserted into the HA locus of the ΔHA mutant. Infected and uninfected cells were labelled with NKp30-Fc, NKp46-Fc and NKp44-Fc. (B) Stainings with anti-HA and NCR-Fc chimeras as in A. Cells were infected with wild-type VV (*gray*), VV:ΔSPI-3 (*black*), VV:ΔHA (*pink*), and VV:ΔHA/ΔSPI-3 (*green*) mutant viruses, respectively. (C) Analysis of NCR binding to HeLa cells infected with VV(WR) for 18 h in the absence (*gray lines*) or presence (*black lines*) of 100 µg/ml cytosine arabinoside (AraC). AraC prevents entry into the late phase of the viral replication cycle. Uninfected (*filled curves*) and infected cells were reacted with anti-HA, NKp46-Fc, NKp44-Fc, or chimeric receptors containing the long or the short ectodomain of NKp30 fused to hIgG1-Fc as indicated.

When analyzing VV deletion mutants we observed that deletion of the HA gene in VV resulted in a complete abrogation of the NKp30-Fc and NKp46-Fc surface stainings by infected cells (Figure 2A), indicating that either HA alone represented the ligand for NKp30 and NKp46, or constituted part of a complex ligand structure. Reinsertion of the HA genes from VV or ECTV into this HA-deficient VV mutant rescued the expression of NKp30 and NKp46 ligands in HeLa cells as shown by infection with such revertant viruses (Figure 2A). The efficiency of infection by the HA-deficient mutant virus was monitored by EGFP, which was introduced into the HA locus of VV strain WR, and expression levels of reinserted HA were monitored using the anti-HA mAb VVI-4G9. In accordance with the comparatively weaker staining of ECTV-infected cells (Figure 1B), infection with the VV(WR):ΔHA-HA(ECTV)*flag* revertant virus reconstituted the staining by NKp30-Fc and NKp46-Fc less efficiently than VV(WR):ΔHA-HA(VV)*flag*, suggesting that HA from VV was more efficiently bound by soluble NCR than HA from ECTV. The nonmembrane-bound viral serine protease inhibitor SPI-3 is associated with HA on the plasma membrane of infected cells [42]. To analyze whether SPI-3 contributed to the ligand structure we analyzed the staining of HeLa cells infected with ΔSPI-3 and ΔHA/ΔSPI-3 VV mutants. While the ΔHA/ΔSPI-3 double mutant produced a complete loss of NKp30 and NKp46 staining similar to the ΔHA mutant, deletion of SPI-3 alone had no effect on NCR binding. Thus, SPI-3 does not appear to be part of the NCR ligand structure. Bimodal stainings for HA, NKp30-Fc and NKp46-Fc in Figure 2B were likely due to a non-synchronized VV infection in this experiment resulting in different HA expression levels in two subpopulations. Staining of VV-infected cells by NKp44-Fc was reduced to background levels after infection with HA-deficient VV suggesting that the weak binding of this NCR was also HA-dependent.

To obtain additional evidence for the involvement of HA in binding of NKp30 and NKp46, HeLa cells were infected with VV strain WR in the absence or the presence of the viral replication inhibitor cytosine arabinoside (AraC). AraC treatment efficiently reduced expression of the early and late-phase product HA [43], which was accompanied by a major reduction of the reactivity of infected cells with NKp30 and NKp46 (Figure 2C). Here we compared for the first time the canonical long ectodomain of NKp30 (contained in the 1C7a, b and c isoforms of NKp30) with the short isoform that lacks 25 amino acids within the Ig-like domain (contained in the isoforms 1C7d, e and f). NKp30(short)-Fc showed a weaker reactivity with VV-infected cells than NKp30(long)-Fc, but also its binding was strongly diminished by AraC treatment (Figure 2C).

### Poxviral HA binds NKp30 and NKp46

**Figure 3:**
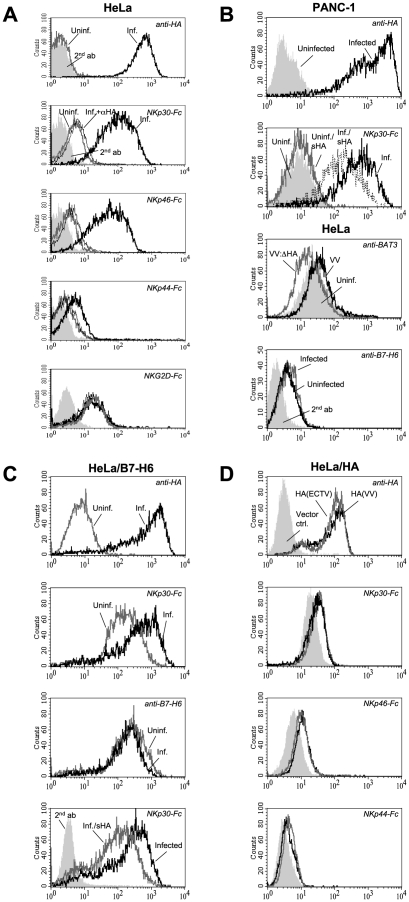
Characterization of HA as ligand for NKp30 and NKp46. (A) VV-infected HeLa cells were stained with anti-HA to monitor infection efficiency. Uninfected (*gray lines*) and infected (*black lines*) were labelled with NKp30-Fc, NKp46-Fc, NKp44-Fc, or NKG2D-Fc without (*thick lines*) and after preincubation of infected cells with an excess of anti-HA mAb (*thin lines*) as indicated. (B, *panels 1-2*) Uninfected and VV-infected PANC-1 cells were reacted with anti-HA to monitor infection efficiency, or with NKp30-Fc, which was either left untreated or preincubated with soluble recombinant HA-His_6_ ectodomains. (B, *panel 3*) Uninfected HeLa cells (*filled histogram*) and HeLa cells infected with either wild-type VV (*black line*) or HA-deficient VV (*gray line*) were stained with chicken anti-BAT3 antibodies and FITC-conjugated goat anti-chicken-Ig secondary antibodies. (B, *panel 4*) Uninfected or VV-infected, normal HeLa cells were stained with anti-B7-H6 mAb to show unaltered basal expression of B7-H6 after infection. (C) VV infection does not upregulate the cellular NKp30 ligand B7-H6. Uninfected (*gray lines*) and VV-infected (*black lines*) HeLa/B7-H6 transfectants were analyzed with anti-HA, anti-B7-H6, and with NKp30-Fc as indicated (*panels 1-3*). The staining with NKp30-Fc, but not the staining with anti-B7-H6, is increased by VV infection. (C, *panel 4*) B7-H6-transfected HeLa cells were VV-infected and stained with NKp30-Fc fusion proteins that were either untreated (*black line*) or preincubated with soluble recombinant HA-His_6_ (*gray line*). The staining with PE-conjugated anti-hIgG secondary antibodies is shown as filled histogram. (D) HeLa cells transfected with HA cDNAs derived from VV(WR) (*black lines*) and ECTV(MP-Nü) (*gray lines*) were stained with anti-HA, NKp30-Fc, NKp46-Fc and NKp44-Fc as indicated. HeLa cells transfected with empty pcDNA3.1(+) expression vector were used for control (*filled histograms*).

As shown in Figure 3A, the preincubation of VV-infected HeLa cells with an excess of the anti-HA mAb VVI-4G9 fully blocked the stainings with NKp30-Fc and NKp46-Fc soluble receptors. Also, the weak binding of NKp44-Fc was cancelled by anti-HA. The binding of NKG2D-Fc, which was used as a control, was not affected by preincubation with anti-HA (Figure 3A). In the same line, preincubation of NKp30-Fc with soluble monomeric HA ectodomains from VV reduced their reactivity with VV-infected PANC-1 cells (Figure 3B). This is probably due to complex formation and saturation of HA binding sites in NKp30-Fc chimeras preventing subsequent binding to cell-expressed HA. Furthermore, preincubation of NKp30-Fc with soluble HA-V5-His_6_ reduced the staining of HeLa cells stably transfected with the cellular NKp30 ligand B7-H6 [28] (Figure 3C). This result suggests that soluble HA bound to NKp30-Fc chimeras and blocked binding sites for B7-H6 within NKp30 ectodomains.

While VV infection of HeLa/B7-H6 transfectants resulted in an enhanced labeling with NKp30, the B7-H6 surface expression of infected cells, which was detected with a novel B7-H6 reactive mAb, was even slightly reduced (Figure 3C). Untransfected HeLa cells only weakly reacted with the anti-B7-H6 mAb, and this staining was not influenced by VV infection (Figure 3B). These results indicate that poxviral HA and B7-H6 molecules are independent ligands for NKp30. The nuclear protein BAT3 has been reported to mediate NKp30 binding after stress-induced transfer to the cell surface [27]. While we detected a slightly increased surface staining for BAT3 in VV-infected HeLa cells, the BAT3 staining of VV:ΔHA-infected cells was reduced (Figure 3B). Thus, a potential BAT3-mediated triggering of NKp30 is unlikely to explain the enhanced cytolytic activity against VV:ΔHA-infected cells (see below).

To study NCR binding to poxviral HA independent of other viral proteins, HeLa cells were stably transfected with HA(VV) and HA(ECTV) genes (Figure 3D). We observed increased binding of NKp30-Fc and NKp46-Fc to HA-expressing HeLa cells. The enhancement of NCR binding was, however, limited, which was likely due to ∼10 times lower cell surface expression levels of transfected HA as compared with VV-infected cells.

In enzyme-linked immunosorbent assays (ELISA), NKp30-Fc and NKp46-Fc also reacted with plate-bound VV particles in an HA-dependent manner but did not recognize HA-deficient VV particles (Figure S2). Moreover, soluble recombinant HA(VV) and HA(ECTV) molecules adsorbed to ELISA plates were recognized by NKp30-Fc and NKp46-Fc, and this reaction could be blocked with anti-HA. NKp30-Fc consistently labeled plate-bound HA 1.5–2-fold stronger than equal amounts of NKp46-Fc (Figure S2 and data not shown), suggesting that NKp30 had a higher affinity for HA than NKp46. The ELISA results fully supported the notion that HA serves as NCR ligand structure.

### Role of NCR-linked glycans and glycans expressed on VV-infected and uninfected cells

**Figure 4:**
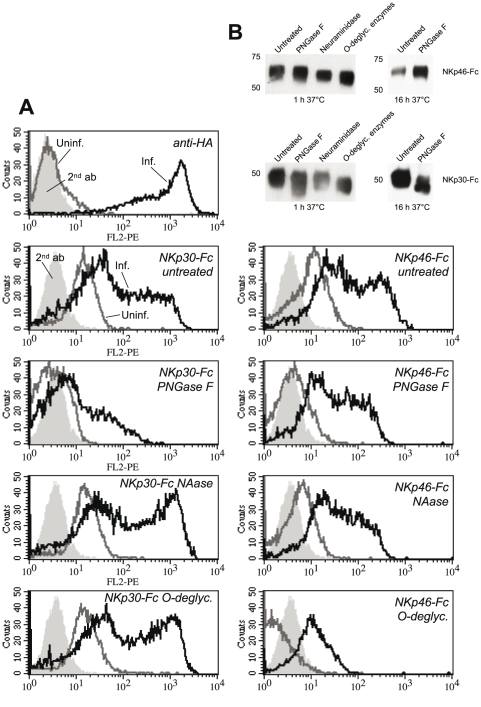
Deglycosylation differentially influences the binding of NKp30-Fc and NKp46-Fc. (A) Uninfected HeLa cells and VV(WR)-infected HeLa cells were stained with anti-HA to monitor infection efficiency. Uninfected (*gray lines*) and infected (*black lines*) were labelled with NKp30-Fc (*left column*) and NKp46-Fc (*right column*) fusion proteins, that were *N*-deglycosylated *in vitro* using protein *N*-glycanase F (PNGase) for 16 h, or treated with α2-3,6,8,8-neuraminidase (NAase) for 1 h, or with a cocktail of *O*-deglycosylating enzymes (α2-3,6,8,8-neuraminidase, β1,4-galactosidase, endo-α-*N*-acetylgalactosaminidase, β1-2,3,4,6-*N*-acetylglucosaminidase) for 1 h at 37°C, respectively. (B) Western blots of enzymatically deglycosylated NKp46-Fc and NKp30-Fc fusion proteins that were detected by NKp30- and NKp46-specific mAbs.

An *O*-linked carbohydrate attached to Thr 225 has been described to regulate the binding of NKp46 to influenza virus HA [44]. Moreover, desialylation abolished the binding of NKp46 and NKp44 ectodomains to Sendai and Newcastle disease virus hemagglutinin-neuraminidase [30], [31], [33]. To better understand molecular mechanisms involved in the recognition of VV-infected cells by NKp30 and NKp46, we studied the involvement of *N*- and *O*-linked glycans attached to NKp30 and NKp46. After binding to protein A Sepharose beads, NKp30-Fc and NKp46-Fc proteins were treated with *N*-deglycosylating PNGase F, a cocktail of *O*-deglycosylating enzymes, or with neuraminidase alone (Figure 4B), and used for the staining of uninfected and VV-infected HeLa cells. As shown in Figure 4A, digestion of potential *O*-glycans in the membrane-proximal domain of NKp46 [44], [45] strongly reduced the binding of NKp46-Fc to VV-infected and uninfected HeLa cells. Desialylation and *N*-deglycosylation only slightly reduced the staining intensity. By contrast, binding of NKp30 to VV-infected cells, for which 2 *N*-glycans but no *O*-glycan have been predicted [46], seems to depend on *N*-glycosylation as the staining was markedly reduced by PNGase F treatment (Figure 4A). Treatment with neuraminidase alone, or the *O*-deglycosylating enzyme cocktail (containing neuraminidase), rather had an enhancing effect on NKp30-Fc binding. We conclude that different types of glycans are involved in HA binding to NKp46 and NKp30, and that these binding modes differ from the sialic acid-dependent binding of NKp46 and NKp30 to other viral ligands as mentioned above.

Natural cytotoxicity receptors have cellular ligands on carcinoma and leukemia cells which are, however, expressed in low quantities on the cell surface of most cell lines. In addition to the proteinaceous NKp30 ligand, B7-H6, heparan sulfate proteoglycans have been implicated in the binding of NKp30, NKp46, and NKp44 [24]-[26]. Furthermore, target cell hyaluronan has been discussed as ligand structure for NKp46 [47]. In order to investigate the potential contribution of glycan structures to the formation of cellular *versus* viral ligands, we enzymatically deglycosylated the cell surface of uninfected and VV-infected HeLa cells. Enzymatic treatments of live cells followed by staining with NCR-Fc chimeras confirmed an important role of proteoglycans as well as of *N*-linked glycans in the formation of cellular ligands for NKp30, NKp46 and NKp44 (Figure S3). In addition, *O*-linked glycans appeared to contribute to cellular ligand structures recognized by NKp44. After VV infection, however, digestion of proteoglycans led to an enhanced binding of NKp30 and NKp46. Removal of anionic proteoglycans may have unmasked counter-receptors for NKp30 and NKp46 to some extent. *O*-linked glycans contributed to the enhanced binding of NKp30 and NKp46 after VV infection since *O*-deglycosylation reduced this binding (Figure S3). Together with the results presented in Figure 4, these findings suggest that glycan/glycan interactions are involved in the recognition of VV-infected cells, and in particular of HA, by NKp30 and NKp46, while exact mechanisms remain to be explored.

### Analysis of NKG2D ligands

Previously it has been reported that the expression of ligands for the activating NK receptor NKG2D was not affected by VV infection of human fetal foreskin fibroblasts [18]. In this work, we essentially confirmed this finding by using NKG2D-Fc chimeric soluble receptors and VV-infected HeLa cells. A detailed analysis of the NKG2D ligands MICA, MICB, ULBP1, ULBP2, ULBP3, and ULBP4 revealed, however, previously unnoted differential down- or upmodulation of these ligands (Figure S4). In contrast to VV, infection of HeLa with ECTV resulted in clearly reduced expression levels of the sum of NKG2D ligands as detected by human NKG2D-Fc (Figure S4), and is thus at variance with a recent study demonstrating upregulation of NKG2D ligands upon ECTV infection of mouse cells [19].

### VV infection and HA expression modulates NK lysis susceptibility and cytokine secretion

**Figure 5:**
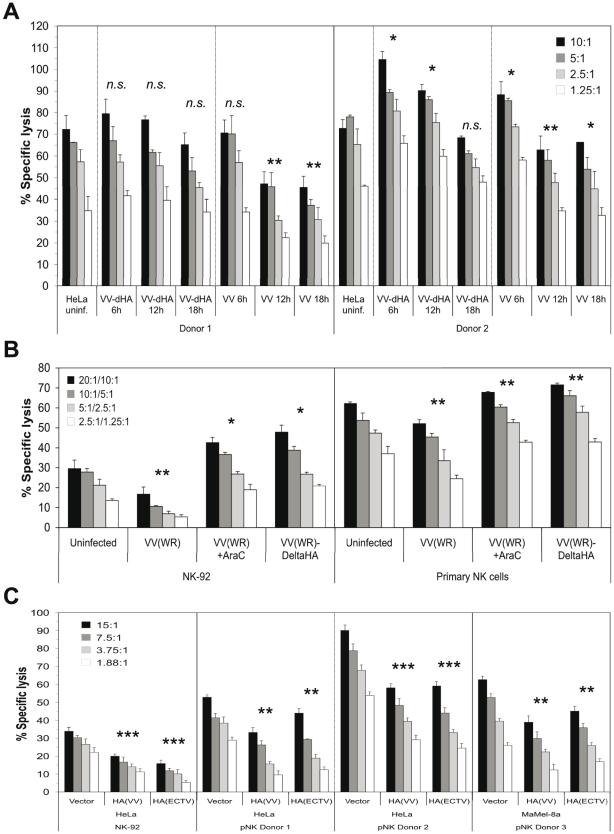
Reduced lysis susceptibility of target cells after infection with wild-type VV. (A) Cytotoxicity assay using primary NK cells from two different donors as effectors and HeLa cells as targets. HeLa cells were either left uninfected or infected with wild-type VV(WR) or HA-deficient VV(WR) for 6 h, 12 h and 18 h, respectively, and labeled with ^51^Cr. Different effector-to-target cell (E:T) ratios are shown. HA surface expression of VV-infected cells, NCR-Fc binding to wild-type VV and VV:ΔHA infected cells as well as the expression levels of NKp30, NKp46, NKp44 and NKG2D on NK cells used in this experiment are shown in Figure S5. (B) Cytotoxicity assay using the NK cell line NK-92 and primary NK cells from two different donors as effectors. HeLa targets were infected with either wild-type VV(WR) in the absence or presence of 100 µg/ml AraC, or with VV(WR):ΔHA mutant virus. HA surface expression and NCR-Fc binding in this experiment is presented in Figure 2C. Different E:T ratios for NK-92/primary NK cells are shown. (C) Cytotoxicity assay using HeLa vector control, HA(VV) and HA(ECTV) transfectants as targets, and NK-92 as well as primary NK cells (pNK) from two donors as effectors. The surface expression levels of transfected HA molecules are shown in Figure 3D. In addition, Ma-Mel-8A transiently transfected with the empty pcDNA3.1(+) expression vector, pcDNA3.1(+)/HA(VV) and pcDNA3.1(+)/HA(ECTV) were used as targets with primary NK cells from a third donor as effectors. For statistical comparisons of virus-infected/HA-transfected samples *versus* uninfected/untransfected samples Student's *t*-test (paired, 2-tailed) was used, *n.s*., not significant *, p<0.05, **, p<0.01, ***, p<0.001.

Next we assessed the functional impact of VV infection on the susceptibility to lysis by primary NK cells from different donors. This is a complex issue due to the involvement of several activating and inhibitory receptors on responding NK cells. A representative lysis assay shows the lysis of VV(WR)- and VV(WR:ΔHA)-infected HeLa cells side by side and after different periods of VV infection (Figure 5A). HA expression in the course of infection and, in case of VV:ΔHA, expression of EGFP were monitored along with the infection time-dependent binding of NKp30-Fc and NKp46-Fc (Figure S5A). NK cells from donor #2 expressed higher levels of NKp30, NKp46 and NKp44, but lower levels of NKG2D, than NK cells from donor #1 (Figure S5B). This phenotype correlated with a more efficient lysis of uninfected and VV-infected HeLa cells by NK cells from donor #2 (Figure 5A, right part). In this, as well as several other NK cell assays with similar outcome, we detected differences in the lysis of target cells infected with either wild-type VV or HA-deficient VV. At late time points of infection (12 h, 18 h), when HA expression was high (Figure S5A), wild-type VV infection resulted in an inhibition of lysis as compared with uninfected or VV:ΔHA-infected targets, suggesting that high HA expression levels had an inhibitory rather than an activating effect on NK cells. At 6 h post infection with wild-type VV, and at 6 and 12 h post infection with VV:ΔHA, we observed a slightly enhanced lysis susceptibility compared with uninfected targets, suggesting that HA-independent early viral factors promoted lysis susceptibility. These assumptions were confirmed in experiments using AraC to block the entry into the late phase of viral replication. AraC treatment during VV infection diminished HA expression and NCR binding (see Figure 2C). While VV infection resulted in a reduced lysis of HeLa cells by the human cell line NK-92 and by primary NK cells, infection with VV:ΔHA or infection with VV in the presence of AraC enhanced the lysis susceptibility as compared with uninfected targets (Figure 5B).

To further investigate the potential inhibitory function of poxviral HA on NK cells, we utilized HeLa and MaMel-8A cells transfected with either HA(VV) or HA(ECTV) in cytotoxicity assays with NK-92 or primary NK cells from different donors. As compared with vector control transfectants, ectopic expression of HA(VV) or HA(ECTV) by these tumor cell lines (see Figure 3D) correlated with a reduced kill by NK-92 and primary NK cells (Figure 5C). In the same line, inclusion of soluble recombinant HA(VV) in the cytotoxicity assay reduced the lysis of uninfected and VV-infected HeLa cells (Figure S6A). Conversely, the preincubation of VV-infected, but not uninfected, HeLa cells with anti-HA mAb enhanced their lysis by primary NK cells (Figure S6B). A partial rescue of the lysis susceptibility of VV-infected targets was achieved when the target cells were pre-incubated with soluble NKp30-Fc (Figure S6C), but not with NKp46-Fc (data not shown). Furthermore, a blocking anti-NKp30 mAb added to the assay mixture partially inhibited the lysis of uninfected or VV:ΔHA-infected, but not VV-infected targets (data not shown), suggesting that in the latter case, HA(VV) had outcompeted antibody binding to NKp30 on NK cells.

In addition, we studied the effect of VV infection on the secretion of the effector cytokines TNF-α and IFN-γ by primary IL-2 activated NK cells after cocultivation with VV-infected cells. HeLa stimulator cells were either left uninfected or infected with wild-type VV(WR) or VV(WR):ΔHA, respectively, before UV irradiation to prevent viral spread to responding NK cells. Coculture of NK cells with UV-irradiated, uninfected HeLa cells stimulated the secretion of TNF-α into the culture supernatant, even though they underwent UV-induced apoptosis. VV:ΔHA-infected HeLa also enhanced TNF-α production, whereas VV-infected HeLa cells blocked TNF-α secretion (Figure S7A). Primary NK cells cultured alone, or cocultured with uninfected HeLa cells, produced large amounts of IFN-γ (Figure S7B). Coculture with VV:ΔHA-infected HeLa cells reduced IFN-γ secretion to ∼80% and coculture with wild-type VV-infected HeLa cells to ∼30% of these levels. We conclude that HA-proficient VV is able to inhibit the secretion of effector cytokines by NK cells.

### NCR triggering is differentially affected by HA and VV infection

**Figure 6:**
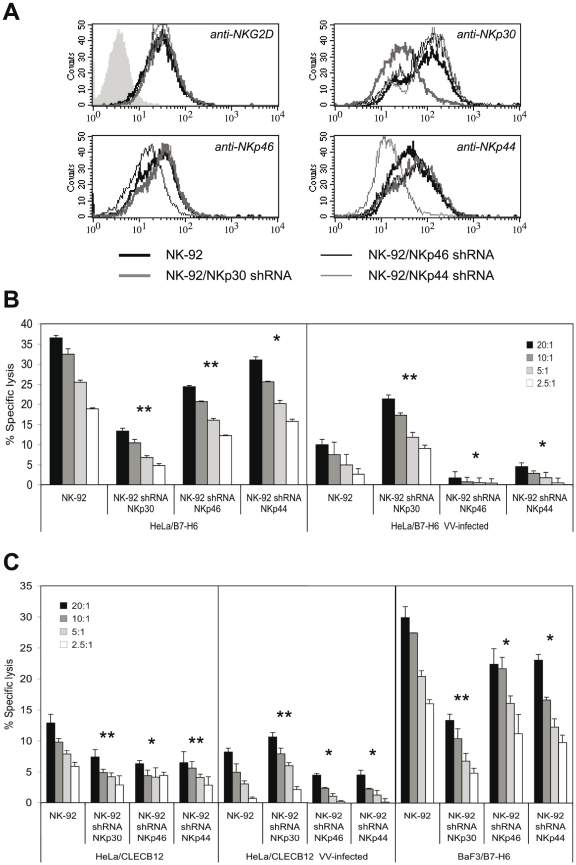
NKp30, NKp46 and NKp44 are differentially involved in the lysis of VV-infected targets. (A) Cytofluorometric analysis of NK-92 cells after retroviral transduction with vectors expressing shRNAs specifically silencing NKp30, NKp46 or NKp44 expression. PE-conjugated anti-NCR mAbs as well as an anti-NKG2D-PE mAb were used for the staining of normal and shRNA-transduced NK-92 cells. (B) Cytotoxicity assay using NCR-silenced and normal NK-92 cells as effectors as indicated. Uninfected and VV-infected HeLa/B7-H6 cells were used as targets. Different E:T ratios are shown. (C) Cytotoxicity assay using NCR-silenced and normal NK-92 cells as effector cells as indicated. Uninfected and VV-infected HeLa/CLECB12 as well as uninfected BaF3/B7-H6 cells were used as targets. Statistical comparison of kills with shRNA-transduced *versus* normal NK-92 cells was done by Student's *t*-test (paired, 2-tailed), *, p<0.05, **, p<0.01.

We sought to characterize the individual contributions of the three NCR to lysis of VV-infected targets. To this end, we transduced the NK cell line NK-92 with lentiviral vectors encoding shRNAs specific for NKp30, NKp46 and NKp44. NK-92 cells express significant levels of NKp30, NKp44 and NKG2D, yet low levels of NKp46 (Figure 6A). The successful downmodulation of NCR expression in stably shRNA-transduced NK-92 cells was demonstrated by staining with anti-NCR antibodies (Figure 6A). In the same way, off-target effects of shRNA transfection on the expression levels of non-targeted NCR and NKG2D were excluded. NK-92 lines with silenced NCR expression were used in cytotoxicity assays with uninfected and VV-infected HeLa/B7-H6 targets (Figure 6B). Consistent with B7-H6 being a major cellular ligand for NKp30, we found that knock-down of NKp30 significantly reduced the lytic capacity against uninfected HeLa/B7-H6. NKp46-silenced NK-92 exerted a reduced kill of uninfected HeLa/B7-H6 suggesting that a NKp46 ligand was expressed by these targets. NKp44 silencing reduced the lytic activity only slightly. VV infection resulted in a strongly reduced lysis of HeLa/B7-H6 by untransduced NK-92 (Figure 6B). Silencing of NKp30 partially rescued this reduced lysis indicating that it was, in part, caused by inhibition through the NKp30 receptor. By contrast, NKp46 shRNA transfection abolished the lysis of VV-infected cells suggesting that cytotoxicity was triggered by NKp46. Also NKp44-silenced NK-92 showed a reduced kill of VV-infected HeLa/B7-H6 targets as compared with regular NK-92. VV-infected and uninfected HeLa/CLECB12 control transfectants as well as BaF3/B7-H6 targets, which were not sufficiently infectable by VV, showed similar patterns of lysis susceptibility to NCR-silenced NK-92 effector cells (Figure 6C).

**Figure 7:**
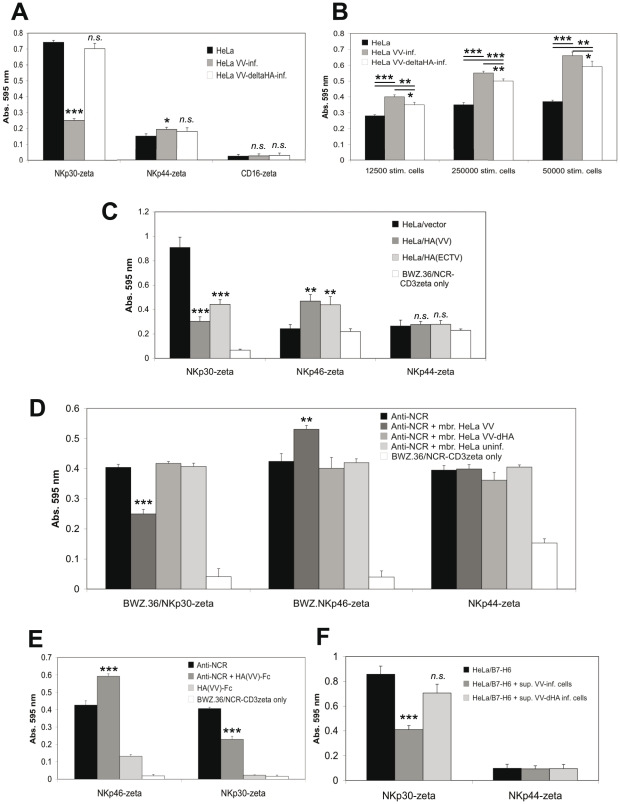
Inhibition of NKp30 triggering and enhancement of NKp46 triggering by poxviral HA. *LacZ*-inducible BWZ.36 reporter cells expressing the ectodomains of NKp30, NKp46, NKp44 or CD16, linked to the transmembrane and cytoplasmic domains of mouse CD3ζ, were used as indicated. Induction of *LacZ* expression is analyzed using the colorigenic substrate CPRG in an ELISA reader as absorption at 595 nm. (A) Reporter cells were cocultured for 18 h with uninfected HeLa cells, or HeLa cells infected with wild-type VV or HA-deficient VV (5×10^4^ stimulator cells/well). Uninfected and infected stimulator cells were pretreated with 254 nm UV light. (B) BWZ.36/NKp46-CD3ζ reporter cells were cocultured for 20 h with uninfected HeLa cells, or HeLa cells infected with wild-type VV or HA-deficient VV using the indicated numbers of stimulator cells. Uninfected and infected stimulator cells were pretreated with UV light. (C) NCR reporter cells were cocultured for 21 h with HeLa cells stably transfected with either the empty pcDNA3.1(+) expression vector, pcDNA3.1(+)/HA(VV) or pcDNA3.1(+)/HA(ECTV), respectively. For a negative control, BWZ.36/NCR-CD3ζ cells were cultured without stimulator cells. (D) NCR reporter cells were cultured for 18 h in 96-wells coated with either anti-NKp30, anti-NKp46 or anti-NKp44 mAbs alone, or together with UV-irradiated membranes prepared from uninfected HeLa cells, or from HeLa cells after infection with either wild-type VV or HA-deficient VV. For a negative control, BWZ.36/NCR-CD3ζ cells were cultured without prior coating of anti-NCR mAbs. (E) NKp30-ζ and NKp46-ζ reporter cells were cultured for 18 h in 96-wells coated solely with anti-NKp30 or anti-NKp46 mAbs, or coated with mAbs together with recombinant soluble HA(VV)-Fc fusion protein. For stimulation of reporter cells, HA(VV)-Fc was also coated alone, and non-coated wells were used for a negative control. (F) NKp30-ζ and, for control, NKp44-ζ reporter cells were cocultured for 18 h with HeLa/B7-H6 cells in the absence or the presence of UV-inactivated supernatants from HeLa cells after infection with either wild-type VV(WR) or VV(WR):ΔHA. All assays were performed in triplicates. For statistical comparisons of virus-infected *versus* uninfected samples (A, B), HA-transfected *versus* untransfected (C), anti-NCR with membranes *versus* anti-NCR without membranes (D), anti-NCR with HA-Fc *versus* anti-NCR without HA-Fc (E), and HeLa/B7-H6 with supernatants *versus* HeLa/B7-H6 without supernatants (F) Student's *t*-test was used, *n.s*., not significant *, p<0.05, **, p<0.01, ***, p<0.001.

NKp30, NKp46 and NKp44 have been reported to occur in molecular complexes facilitating synergistic signaling [48]. This cross-talk might obscure the functional outcome of individual interactions *in trans* between HA and NCR. To selectively study triggering by NKp30, NKp46 and NKp44 receptors we utilized *LacZ*-inducible BWZ.36 NCR-CD3ζ reporter cells as previously published by us [33]. Infection of HeLa cells with VV, but not VV:ΔHA, blocked the basic stimulation of BWZ.36/NKp30-ζ cells by uninfected HeLa cells (Figure 7A). By contrast, VV and, to a lesser extent, VV:ΔHA infection stimulated *LacZ* induction by BWZ.36/NKp46-ζ (Figure 7B), while the influence of VV infection on BWZ.36/NKp44-ζ reporter cells was minor (Figure 7A). We also used HeLa cells stably transfected with HA from VV or ECTV and vector control transfectants as stimulator cells. HA expression inhibited the activation of NKp30-ζ and stimulated the activation of NKp46-ζ cells, whereas we noted no significant HA-specific response by NKp44-ζ cells (Figure 7C). In another approach, we plate-coated anti-NCR antibodies to provide for a basic stimulation of BWZ.36/NCR-ζ reporter cells. Crude plasma membrane preparations from VV-infected, VV:ΔHA-infected and uninfected HeLa cells were added to the assay wells. As shown in Figure 7D, membranes from VV-infected cells interfered with the activation of NKp30-ζ while they acted cooperatively with anti-NKp46 for the activation of NKp46-ζ reporter cells. Membranes from VV:ΔHA-infected and uninfected HeLa cells had no effect. Likewise, soluble recombinant HA(VV)-Fc partially blocked the activation of NKp30-ζ cells by anti-NKp30, but enhanced the activation of NKp46-ζ cells (Figure 7E). Surface-adsorbed HA(VV)-Fc stimulated NKp46-ζ, but not NKp30-ζ or NKp44-ζ reporter cells (Figure 7E). An inhibition by supernatants from VV-infected cells was also seen when HeLa/B7-H6 cells were used for the activation of NKp30-ζ reporter cells (Figure 7F). Taken together, the results obtained with NCR reporter cells demonstrated that HA had an activating effect for NKp46 whereas it blocked activation through NKp30.

## Discussion

Natural killer cells represent an important element in protective immune responses against vaccinia and mousepox virus. This is mainly evidenced by mouse models in which NK cells were depleted by anti-asialo-GM1 antibodies [6], [11], [13], [14], [19], [49]. Low NK cell cytotoxicity in humans is associated with an increased susceptibility towards severe and recurrent herpesgroup virus infections [50], whereas little is known about the role of human NK cells in the defense of poxviral infections.

VV infection sensitizes human tumor cell lines and autologous T cells for NK-mediated lysis *in vitro* [51]. More recently, it has been shown that VV-infected human fibroblasts are recognized by NK cells through the NCR NKp46, NKp44 and NKp30 [18]. VV-induced NCR ligand structures were, however, not identified in that study. Genetically engineered forms of the highly immunogenic poxvirus VV have been successfully employed for vaccinations of humans [3], [52]. Moreover, attenuated VV strains are presently considered for oncolytic virotherapy in humans [41]. Therefore, it is of importance to identify NK cell ligand structures induced by poxviruses, particularly in the course of VV infection.

In this study we have identified VV hemagglutinin as a novel virus-encoded ligand for the activating NK cell receptors NKp30 and NKp46. Several lines of evidence presented herein led to this conclusion; i, the induction of ligand structures on VV-infected human carcinoma cells, as detected by staining with soluble recombinant NKp30 and NKp46 receptors, was fully abolished after deletion of HA in the viral genome and reconstituted after reinsertion, ii, anti-HA antibodies blocked the enhanced staining of VV-infected cells by NKp30- and NKp46-Fc, iii, soluble recombinant HA interfered with the binding of NKp30 to VV-infected cells, to VV particles, and to its cellular ligand B7-H6; and iv, HA-transfected cells showed an increased reactivity with NKp30-Fc and NKp46-Fc. Furthermore, the binding of NKp30 and NKp46 to plate-bound VV, but not VV:ΔHA particles, as well as the blocking effects of anti-HA antibodies and soluble recombinant HA, confirmed the staining of VV-infected cells. In the same line, infection of HeLa cells with the mouse poxvirus ECTV resulted in a significant induction of ligands for NKp30 and NKp46. HA(ECTV) reconstituted NKp30 and NKp46 binding either after infection with a VV:ΔHA:HA(ECTV) virus revertant (see Figure 2A) or in HeLa cells stably expressing HA(ECTV) (see Figure 3D). Our results indicate that the HA molecule from ECTV also serves as a ligand for human NKp30 and NKp46, however, a HA deletion mutant of ECTV still needs to be investigated. Mouse NK cells express NKp46 [53], but not NKp30 [54]. It will be interesting to analyze in future studies whether ECTV-infected cells are recognized by the murine homologue of NKp46. Furthermore, the possible interactions of human NKp30 and NKp46 with variola virus HA, whose extracytoplasmic domain shares ∼84% amino acids with vaccinia HA, will be of interest since variola is able to establish a systemic infection of immunocompetent human hosts and might thus be able to overcome a first-line defence by NK cells more readily.

In accordance with a previous study using human fibroblasts for infection [18], we detected an induction of NKp44-Fc binding after infection with VV or ECTV (see Figure 1), which was, however, weak as compared with the staining of infected cells by NKp30-Fc and NKp46-Fc. This slightly increased binding of NKp44-Fc was not observed when cells were infected with the HA-deficient mutants VV:ΔHA and VV:ΔHA/ΔSPI-3. Therefore, it seems possible that poxviral HA represents a minor ligand for NKp44 as well. Binding of NKp44-Fc to viral particles in ELISA and to HA-transfected HeLa cells was, however, negligible. The latter result may be explained by the comparatively low expression levels of HA(VV) and HA(ECTV) on the surface of HA-transfected cells.

In keeping with previous work [18], the entirety of NKG2D ligands as detected by NKG2D-Fc soluble receptors were not significantly altered after VV infection. Upon detailed inspection we noted, however, a differential behavior of the six NKG2D ligands tested, with ULBP1, ULBP2 and ULBP4 being slightly upregulated, MICA and ULBP3 unaltered, and MICB slightly downregulated. Presently, we have no explanation for this complexity. Here we show that ECTV infection leads to a partial loss of staining with human NKG2D-Fc which is reflected in a decreased reactivity of antibodies against MICB, MICA and ULBP3 with ECTV-infected human cells. This finding contrasts with a recent report showing enhanced expression of mouse NKG2D ligands upon ECTV infection of mouse embryo fibroblasts and enhanced NKG2D-mediated cytotoxicity [19]. It is unexpected as humans are not the natural host for ECTV.

Recently, the B7-H6 molecule has been identified as cellular ligand for NKp30 [28]. The B7 family member B7-H6 contains a membrane-distal IgV-like domain and a membrane-proximal IgC-like domain. VV(HA) and NKp30 both contain a membrane-distal IgV-like domain linked to the membrane through a short glycosylated stalk region [46], [55]. HA from ECTV and VV show 92.5% sequence homology within the N-terminal Ig-like domain. In light of these structural similarities it seems reasonable to assume that homotypic interactions within IgV-like domains play a role in the binding of NKp30 to B7-H6 and poxviral HA, respectively. As no Ig superfamily member has so far been reported as ligand structure for NKp46, the interaction of NKp46 with poxviral HA remains elusive at present.

Unexpectedly, we observed that after 12–18 hrs of VV infection, target cells became less susceptible to lysis by polyclonal NK cells or by the NK cell line NK-92. At early time points of infection, when HA expression was low or when the late viral phase was blocked by AraC, the susceptibility of infected HeLa cells to NK lysis was, however, slightly increased. Regarding these modulating effects of VV infection, we noted minor differences among primary NK cells from different donors. Since infection with the HA-deficient virus did not reduce lysis susceptibility as compared with uninfected targets, we reasoned that HA, which is mainly expressed as a late-phase product [43], had an inhibitory effect on NK cells, whereas some early phase products may exert a stimulatory effect on NK cells. This infection-time dependent modulation of NK activation and other target cell-mediated effects might explain why in a previous report using slightly shorter periods of VV infection and human foreskin fibroblasts as targets, an enhanced lysis but no inhibition was noted [18]. This contention is corroborated by our results (see Figure S1) showing that, using the same infectious dose and infection time, VV-infected human fetal lung fibroblasts and particularly human foreskin fibroblasts expressed much lower levels of HA than HeLa cells, corresponding to lesser enhancements of the stainings by NKp30-Fc and NKp46-Fc. In agreement with our results, in the mentioned study NK lysis was enhanced after infection with the HA-deficient VV strain IHD-W as compared with its HA-competent counterpart IHD-J [18], [56].

The inhibitory effect of HA on NK lysis was further substantiated in this study by using i, VV(WR)- and VV(WR):ΔHA-infected targets for NK-92 effectors, ii, using transfectants expressing HA from VV or ECTV, and iii, using anti-HA antibodies and soluble NKp30-Fc to enhance the lysis of VV-infected cells (see Figures 5 and S6). The poxviral HA(A56R) molecule, which has no lectin-binding properties and is not essential for viral infection [56], is strongly expressed on the surface of VV-infected cells and in a major proportion of EEV membranes [57]. Together with the HA-associated SPI-3 serpin (K2L), HA blocks the entry of superinfecting virus into VV-infected cells and syncitia formation [58], [59]. HA is a proviral virulence factor as HA-competent virus replicates much more rapidly in hosts than HA-deficient virus [60], [61]. Our herein reported finding that NK cell-mediated lysis of wild-type VV-infected and HA-transfected target cells was reduced is consistent with the higher virulence of HA-proficient VV and constitutes a novel immune escape mechanism of VV, and possibly other poxviral family members, during the late phase of infection. Our data also showed that SPI-3 was not required for the inhibitory effect of HA (see Figure 2).

Coculture of preactivated primary NK cells with VV-infected, UV-irradiated tumor cells resulted in a significant reduction of IFN-γ and TNF-α secretion compared to stimulation by uninfected, UV-irradiated cells. This functional blockade of NK cytokine secretion was considerably more pronounced in the presence of HA-competent than HA-deficient VV, suggesting that HA exerted an inhibitory effect. As the inhibition of cytokine secretion *in vitro* may require a direct contact between infected cells and NK cells, it does not exclude a systemic activation of NK cells by type I interferons in secondary lymphoid organs or other non-infected tissues [17], and is therefore not in conflict with protective NK-mediated IFN-γ responses during poxviral infection as reported earlier [6], [14], [15].

Using NK-92 effectors with selectively silenced NCR expression and *LacZ*-inducible, NCR-CD3ζ reporter cells we dissected differential effects of VV infection and HA expression on NKp30 and NKp46 triggering. Collectively, our results clearly document an inhibition of NKp30-mediated activation and a stimulation of NKp46-mediated activation. This was observed using VV-infected cells as targets for NCR-silenced NK-92, and also when NCR-CD3ζ reporter cells were cultured with UV-inactivated infected cells, membranes or supernatants derived from infected cells, HA(VV) and HA(ECTV) transfectants, or soluble recombinant HA, respectively. Presently, we do not understand how poxviral HA exerts its inhibitory effect on NKp30-mediated NK activation, regardless of NKp30 being present in its natural conformation on NK cells or being expressed as a CD3ζ-linked dimer in BWZ.36 reporter cells. In preliminary experiments using primary NK cells, we failed to detect a reduced CD3ζ association with NKp30 after interaction with VV-infected Hela cells. Such a mechanism has been proposed for the inhibitory effect of HCMV pp65 protein on NK cells activation through NKp30 [34]. We rather assume that poxviral HA binds to NKp30 in such a way that *cis*-interactions of NKp30 with itself or other proteins and thereby induced conformational changes required for signal transduction may be prevented. Conversely, the interaction of HA with NKp46 appears to be productive as NKp46-CD3ζ reporter cell triggering is stimulated. Because the net effect of NK cell interaction with VV-infected cells or soluble recombinant HA appears to be inhibitory, we assume that interaction of HA with NKp30 functionally dominates the interaction with NKp46.

As cells infected with VV(WR):ΔHA showed an increased lysis susceptibility (see Figure 5) we searched for potential HA-independent activating ligands. The known NKp30 ligand B7-H6 could be ruled out (see Figure. 3), and we also could not pin-point MHC class I downregulation as being responsible for enhanced NK activation (unpublished results). Consistent with earlier work [62], regulators of complement activation, CD46, CD55 and CD59, were slightly enhanced after VV and VV:ΔHA infection (unpublished results). CD59 might thus play a role for VV-enhanced, HA-independent NK cell activation [63]. The surface expression of LFA-1, LFA-3, CD44, CD24, and in particular of CD62L molecules, was induced after VV and VV:ΔHA infection of HeLa cells (unpublished results). The functional significance of these findings in terms of augmented NK cell adhesion to VV-infected cells remains open at present.

In summary, the herein presented results characterize poxviral, and in particular vaccinia viral, hemagglutinin as novel ligand structures for the natural cytotoxicity receptors NKp30 and NKp46, with a possible additional involvement of NKp44. While NCR reporter cells indicated a blockade of NKp30 triggering through HA and a stimulation of NKp46 triggering, an inhibitory effect of VV infection on lysis susceptibility of cancer cells became dominant at late time points of infection when HA expression was pronounced. In poxvirus oncolytic therapy, NK cell-mediated cytotoxicity is discussed to play an important role. Our findings would support the use of HA-deficient VV variants in therapeutic approaches employing oncolytic poxviruses.

## Materials and Methods

### Cell lines

The human cervix carcinoma HeLa, the human pancreatic adenocarcinoma line PANC-1, the murine mastocytoma line P815, and the fetal lung fibroblast line MRC-5 were obtained from the American Type Culture Collection (ATCC). The human foreskin fibroblast line VH7 has been described [64]. B7-H6::GFP transduced HeLa and BaF3 cells are described in Supporting Information Text S1. HeLa/CLECB12 transfectants have been described [65]. The metastatic melanoma cell line Ma-Mel-8a [66] was a kind gift from Dr. A. Paschen, Department of Dermatology, University Clinics Essen. The murine lung carcinoma line TC-1/A2 [67] was provided by Dr. A. Cid, DKFZ, Heidelberg. Cell lines were cultured in RPMI-1640 (Invitrogen, Karlsruhe, Germany) supplemented with 2 mM glutamine and 10% fetal calf serum. NK-92CI [68] is grown in MEM α medium supplemented with 2 g/l NaHCO_3_, 12.5% fetal calf serum, 12.5% horse serum, penicillin/streptomycin, 1% L-glutamine, and 50 µM 2-mercaptoethanol. NK-92CI (hereafter referred to as NK-92) is a human natural killer tumor cell line that has been transfected with human IL-2 cDNA and grows independently of IL-2.

### Plasmids and transfections

The hemagglutin genes of VV strain WR and ECTV strain MP-Nü were cloned from mRNA of virus-infected cells using following primers HA(WR)-5′: GAGAAAGGTACCAGATCTCTAATATGACACGATTACCAATACTTTTG, HA(ECTV)-5′: GAGAAAGGTACCAGATCTTAATATGGCACGATTGTCAATACTTTTG, and HA-3′: CTCTCGAGCTCGGATCCGACTTTGTTCTCTGTTTTGTATTTACG, and inserted in frame with the FLAG epitope in the vector p7.5k-131A-FLAG. From these plasmids the coding sequences for HA(VV)*flag* and HA(ECTV)*flag* were subcloned in pcDNA3.1(+) (Invitrogen). 2.5×10^5^ HeLa cells cultured in 6-well plates were transfected with 4 µg pcDNA3.1(+)/HA(VV)*flag*, pcDNA3.1(+)/HA(ECTV)*flag* or empty pcDNA3.1(+) vector and 10 µl lipofectamine 2000 (Invitrogen) according to the manufacturer's instructions. After 2 days, cells were selected with geneticin (1 mg/ml in Dulbecco's phosphate-buffered saline [DPBS]; Sigma-Aldrich, Taufkirchen, Germany) and, after cell sorting for high HA expression, maintained at 0.5 mg/ml geneticin.

The cloning and expression of B7-H6 cDNA is described in Supplemental Materials and Methods (Text S1).

### Preparation of VV stocks

The VV strains Western Reserve (WR), ectromelia virus (ECTV) strain MP-Nü (kindly provided by Dr. H. Ellerbrock, Robert-Koch-Institut, Berlin, Germany) were used. VV(WR) was parent of the mutants VV(WR):ΔHA, VV(WR):ΔHA-HA(VV)*flag* and VV(WR):ΔHA-HA(ECTV)*flag* (*see* Construction of VV HA mutants). Crude stocks of VV and ECTV were prepared from supernatants of sonicated infected cells. Cell debris were removed by centrifugation at 1,680 x g for 10 min. To produce purified VV stocks supernatants of infected cells were subjected to two additional centrifugation steps (1,680 x g for 10 min), followed by centrifugation through a sucrose cushion (36% sucrose, 31,000 x g, 90 min). VV titers were determined by a standard plaque assay using CV-1 cells.

The SPI-3 and HA-deficient VV mutants VV-T7ΔSPI-3, VV-T7ΔA56 and VV-T7ΔSPI-3/ΔA56 have been described [58] and were amplified and purified as described above. Following infection of HeLa cells, HA and/or SPI-3 deficiency were confirmed using the HA-specific mAb VVI-4G9 and the SPI-3-specific mAb 4A11-4A3 (*see* Antibodies).

### Construction of VV HA mutants

The VV HA deletion mutant was cloned by replacing the HA gene with an EGFP cassette through homologous recombination. The 5′ and 3′ flanking regions of the VV HA gene were cloned in the pUC18 vector using the following primer pairs, WR180-*Kpn*I: GAGGGTACCCTCGTTCTAATTGTGGGGGACTG / WR181ATG-*Nco*I: CTCGGATCCGTATTGGTAATCGTGCCATGGATTAGTATAAAAAGTG, and WR181-*Bam*HI: GAGGGATCCGCGGCCGCATTTTTGACTTACATAAATGTCTGGGATAG / WR182-*Sph*I: CTCGCATGCAATACATTCTAATACGGTCCTGTAGTATCTG. An EGFP cassette was cloned between the flanking regions. For HA deletion, African green monkey CV-1 cells (ATCC CCL-70) were infected with wild-type VV (multiplicity of infection [m.o.i.] 0.05) and after 2 h transfected with the pUC plasmid containing the EGFP gene between HA flanking regions using the SuperFect transfection reagent (QIAgen, Hilden, Germany). Infected cells were harvested 2 days later and lysates produced by freezing/thawing. CV-1 cells were infected with the lysate in limiting dilution. EGFP-expressing viruses were selected in two rounds of infection. HA deficiency was confirmed by cytofluorometric analysis using the anti-HA mAb VVI-4G9.

FLAG epitope-tagged HA genes from VV and ECTV were reinserted into the thymidine kinase (*TK*) locus of the VV(WR):ΔHA mutant. For homologous recombination, p7.5k-HA(WR)*flag* and p7.5k-HA(ECTV)*flag* plasmids containing HA codings sequences under control of the *TK* promoter and homologous flanking regions of the *TK* gene were used. The recombinant viruses VV(WR):ΔHA-HA(WR)*flag* and VV(WR):ΔHA-HA(ECTV)*flag* were selected by infecting 143 *tk^−^* cells in the presence of bromodeoxyuridine.

### Viral infection of tumor cells

Tumor cells were seeded in 6-well plates at ∼1×10^6^ cells/well. Adherent cells were washed once in D-PBS and m.o.i. 4 of VV or ECTV were added in 500 µl RPMI-1640 without FCS for 1–2 h at 37° in a CO_2_ incubator. Medium was replaced by 2.5 ml complete RPMI-1640 medium in incubated at 37°C for up to 20 h before harvesting in D-PBS/0.05% trypsin/5 mM EDTA or enzyme-free cell dissociation buffer (Invitrogen). In some experiments, cytosine D-arabinofuranoside (AraC) (Sigma-Aldrich), which prevents viral DNA replication and synthesis of late-phase proteins, was present throughout the infection period at 100 µg/ml.

### Soluble recombinant HA and NCR-Fc fusion proteins

The ectodomains of HA from VV(WR) and ECTV were amplified by using the primer pairs HA(WR)-5′: GAGAAAGGTACCAGATCTCTAATATGACACGATTACCAATACTTTTG / sHA(WR)-3′: GAGGAATTCCTACAAAGTCCTTGGTTTTATAATTGC and HA(ECTV)-5′: GAGAAAGGTACCAGATCTTAATATGGCACGATTGTCAATACTTTTG / sHA(ECTV)-3′: GAGGAATTCTCTGCAAAGTCTTTAGTACTATACTTACCTAT and subcloned in the vector pGene/V5-His. From these plasmids, the coding sequences for V5-His_6_-tagged, soluble HA(VV) and HA(ECTV) molecules were subcloned in the plasmid pIRES2-EGFP (BD Biosciences/Clontech, Heidelberg, Germany). To boost gene expression, a β-globin intron II (derived from plasmid pSG5, Stratagene, Amsterdam, The Netherlands) was inserted between the promoter and coding sequences. Supernatants of HEK293T cells transiently transfected using the SuperFect transfection reagent (Qiagen) were harvested and recombinant HA(VV)-V5-His_6_ and HA(ECTV)-V5-His_6_ proteins were purified using His-SpinTrap columns (GE Healthcare). The correct molecular weights of recombinant proteins were verified by Western blot using a V5 epitope-specific mAb (Invitrogen).

To produce recombinant, Fc-linked HA molecules, the ectodomain of VV(WR) (Met_1_-Val_277_) was excised out of pIRES2-EGFP/VV(WR)-V5-His_6_ and cloned in frame with PCR-amplified cDNA coding for the Fc portion of mouse IgG2a (E_232_-K_464_) (accession no. BC031470), using a short EcoRI/BamHI-flanked linker sequence (GILQPGGS) derived from pBluescript KS II+ (Invitrogen). The HA-Fc fusion protein was subcloned into the expression vector pMT2mcs for transient transfection of HEK293T cells and purification by protein A Sepharose CL-4B (GE Healthcare Life Sciences, Freiburg, Germany) as described [33], [69]. The integrity of purified HA-Fc was checked on Western blot using peroxidase-labeled goat anti-mouse IgG/Fc and enhanced chemoluminescence as described [69].

NCR-Fc fusion proteins were produced in HEK293T cells as described [26], [33]. Using the calcium phosphate transfection method, 293T cells were transiently transfected with NKp46-hIgG1-Fc, NKp44-hIgG1-Fc, and NKp30-hIgG1-Fc cDNAs [31] subcloned into the expression vector pMT2+mcs [69]. The ectodomain of the NKp30 isoform 1C7d/e/f with a shorter IgC2-like domain (accession no. Y14768; ΔE65–H89/Q90E with regard to the Ig-like, V-set domain of the long isoform) has been kindly provided by Dr. Ofer Mandelboim, (Hebrew University, Jerusalem, Israel). The short NKp30 ectodomain was also Fc-linked and produced in HEK293T cells. NCR-Fc chimeric proteins were purified using protein A Sepharose CL-4B (GE Healthcare Life Sciences, Freiburg, Germany). The integrity of purified NCR-Ig fusion proteins was confirmed by Western blots using peroxidase-labeled goat anti-human IgG/Fc. Enzymatic *N*-/*O*-deglycosylations of NCR fusion proteins were done after binding to protein A Sepharose beads to allow for enzyme removal by washing. Deglycosylations were carried out as described for VV-infected und uninfected cells (Text S1), except that PBS [pH 6.0] was used as reaction buffer, and PNGase F digestions were done for 1 or 16 h at 37°C. The integrity of fusion proteins and molecular weight shifts after deglycosylation were confirmed by Western blot using mAbs specific for NKp46 and NKp30 and peroxidase-labeled goat anti-mouse IgG secondary antibodies.

### Antibodies

Monoclonal antibodies (mAbs) to VVI-4G9 to and B2D10 to VV hemagglutinin were kindly provided by J.W. Hooper (Virology Division, USAMRIID, Fort Detrick, MD), and H. Shida (Institute for Genetic Medicine, Hokkaido University, Sapporo, Japan), respectively. Rabbit polyclonal antibody to vaccinia virus was from quartett Immunodiagnostika, Berlin, Germany. The SPI-3 reactive mAb 4A11-4A3 has been described [70]. Additional antibodies specific for NK and tumor cell surface proteins and secondary antibodies are described in Supplemental Materials and Methods (Text S1).

### Flow cytometry

For cell surface immunofluorescence stainings, ∼0.5×10^6^ cells were washed once in ice-cold FACS buffer (D-PBS/2% FCS) followed by incubation with a saturating amount of the primary mouse mAb antibody (0.5–1 µg purified antibodies, 100 µl hybridoma culture supernatants) for 45 min on ice. After 2 washes, cells were incubated with PE-labeled goat anti-mouse Ig for 20 min on ice. NCR-Fc fusion proteins (1–2 µg per staining) were complexed with PE-labeled goat anti-human IgG/Fc (Dianova; 1 µl in 100 µl FACS buffer) for 30 min before addition to cells for 45 min on ice. Cells were washed twice with 1 ml FACS buffer and resuspended in 100 µl FACS buffer with 1.3 µg/ml propidium iodide (Sigma-Aldrich) to label dead cells. Cytofluorometric analyses were done using a FACSCalibur flow cytometer and CellQuest software (Becton Dickinson, Heidelberg, Germany). For all FACS stainings, representative examples from at least 3 repeats with similar results are shown.

### Ethics statement

Primary human NK cells were exclusively isolated from buffy coats, which were purchased from the Institute for Clinical Transfusion Medicine and Cellular Therapy, Heidelberg, and had been made anonymous. The Ethics Commission of the University of Heidelberg permitted the use of buffy coats for research purposes without an informed consent by the anomymous blood donor.

### Human primary NK cells

Human polyclonal NK cells were isolated from buffy coats using the NK cell negative isolation kit (Dynal/Invitrogen). 95-99% of the purified NK cells were CD3^−^/CD56^+^. Cells were grown in Iscove's modified Dulbecco's medium (IMDM, Invitrogen), supplemented with 10% human serum, penicillin/streptomycin, 100 U/ml IL-2 (National Institutes of Health cytokine repository), 1 µg/ml phytohemagglutinin P (PHA-P) and irradiated JY lymphoblastoid cells as feeder cells.

### shRNA silencing of NCR

For production of lentiviruses expressing NCR-specific shRNAs, 5×10^6^ HEK293T cells at exponential growth phase (70–80% confluent) in DMEM/10% FCS medium were transfected in a 150 cm^2^ flask by the calcium phosphate precipitation method with the following plasmids: 30 µg pLKO.1 shRNA plasmid (1 µg/µl), 15 µg packaging plasmid (psPAX2, 1 µg/µl), 6 µg VSV-G envelope plasmid (pMD2.G, 0.5 µg/µl). To the plasmid mixture, first 1260 µl H_2_O and 163 µl 2.5 M CaCl_2_, and then 1500 µl 2x HBS (280 mM NaCl, 100 mM HEPES, 1.5 mM Na_2_HPO_4_ [pH 7.12]) were added under vortexing and aeration for 30 sec. The DNA precipitate was added slowly to HEK293T cells. The packaging and envelope plasmids were purchased from Invitrogen. Human pLKO.1 lentiviral shRNA target gene sets for NCR1, NCR2 and NCR3 were from ABgene (Epsom, UK). Clones TRC0000063537, TRC0000063500 and TRC0000063270 yielded the best silencing effects for NKp46, NKp44 and NKp30, respectively.

24 h after transfection, culture supernatants were collected from transfected cells and filtered through a 0.22-µm sterile filter. The medium was replaced and collected again after 48 h. Cell medium was stored in 3 ml aliquots at −80°C. For spinfection of 0.5×10^6^ NK-92 cells, 3 ml of lentivirus-containing medium (with 0.5 µg/ml Polybrene [Sigma-Aldrich]) was used. Transduced cells were selected with 1 µg/ml puromycin. The transduction efficiency was assessed by staining with NCR-specific mAbs. Transduced NK-92 cells were selected with 1 µg/ml puromycin.

### Chromium release assays

Target cells (0.5×10^6^) were labeled in 100 µl assay medium (IMDM with 10% FCS and 1% penicillin/streptomycin) with 100 µCi (3.7 MBq) of sodium chromate [^51^Cr] for 1 h at 37°C. Cells were washed thrice and resuspended at 5×10^4^ cells/ml in 100 µl assay medium. Effector cells were resuspended in assay medium (100 µl/well supplemented with 200 U/ml IL-2) and mixed at different effector to target (E:T) ratios with 5000 labeled target cells/well in a 96-well V-bottom plate. In some experiments recombinant soluble V5-His_6_-tagged HA(VV) or ICOS-L for control was included at ∼1 µg/well. In other experiments, uninfected and VV-infected target cells were preincubated during the labeling time with the anti-HA mAb VVI-4G9, the irrelevant mAb MOPC-21, or NKp30-Fc at ∼2 µg/well. In these experiments, primary NK cell effectors were preincubated with the FcγRIII-blocking antibody CLB-FcR gran/1 (2 µg/1.2×10^6^ effector cells for 20 min). Maximum release was determined by incubation of target cells in 1% Triton X-100 solution. Spontaneous ^51^Cr release was measured by incubating target cells in the absence of effector cells. All samples were done in triplicates. Plates were incubated for 4 h at 37°C. Supernatant was harvested and ^51^Cr release was measured in a γ-counter. The percentage of cytotoxicity was calculated according to the following formula: ([experimental release – spontaneous release] / [maximum release – spontaneous release]) x 100. The ratio between maximum and spontaneous release was at least 3 in all experiments.

### CD3ζreporter cells and *LacZ* assays

The generation of *LacZ*-inducible BWZ.36 reporter cells expressing NKp46-CD3ζ, NKp44-CD3ζ, NKp30-CD3ζ, or CD16-CD3ζ and the procedure of the *LacZ* assay has been described [33]. Briefly, HA-transfected or normal HeLa cells were plated in a 96-well round-bottom plate (1.25–5×10^4^ cells/well). Adherent HeLa cells were infected using m.o.i. 4 in 96-well flat-bottomed cell culture plates and cultured for 20 h in 100 µl RPMI-1640/10% FCS medium followed by UV inactivation (254 nm) of viral particles for 10 min. In some experiments, ELISA plates were coated with 0.5 µg mAbs against NKp30, NKp46 and NKp44 (from R&D Systems) in 0.05 M carbonate buffer (pH 9.6) overnight before washing and addition of reporter cells. In other experiments, ELISA plate wells were coated with ∼1 µg of recombinant soluble HA(VV)-Fc alone or in combination with anti-NCR mAbs. 50 µl culture supernatant from VV(WR)- or VV(WR):ΔHA-infected HeLa cells (m.o.i. 4, 20 h), that had been cleared from cellular debris by centrifugation at 2300 x g for 5 min and inactivated by exposure to 254 nm UV light for 10 min, was added to adherent HeLa/B7-H6 cells in triplicate wells. In another approach, crude membranes were produced from 5×10^6^ VV-infected and uninfected HeLa cells by three cycles of freezing in liquid N_2_ and thawing, followed by clearing of intact cells and nuclei at 1000 x g for 5 min, and pelleting of membranes at 16000 x g for 10 min at 4°C in a microcentrifuge. Crude membranes were resuspended in 250 µl and 10 µl added to assay wells in triplicates.

Adherent tumor cells were co-cultured with 10^5^ NCR-CD3ζ reporter cells for 18 h at 37°C. PMA (phorbol 12-myristate 13-acetate; 5 ng/ml; Sigma-Aldrich) plus ionomycin (0.5 µg/ml; Merck/Calbiochem, Darmstadt, Germany) was used as positive control for the *LacZ* inducibility in reporter cells. The stimulation of NCR-CD3ζ cells was determined by addition of *LacZ* substrate buffer (9 mM MgCl2, 0.15 mM CPRG, 100 mM 2-ME, 0.125% Nonidet P-40 in PBS [pH 7.5]) for 4 h at 37°C. The cleaved CPRG was measured in an ELISA reader with an absorbance at 595 nm with 630 nm as the reference wavelength. The data are shown as means of triplicate values and s.e.m. from representative examples of three similar experiments.

Additional experimental methods can be found in Supplemental Materials and Methods (Text S1).

## Supporting Information

Figure S1 **Induction of NKp30 and NKp46 ligands on VV-infected human fibroblasts.** Human MRC-5 fetal lung fibroblasts (A) and human VH7 foreskin fibroblasts (B) were infected with VV(WR) using m.o.i. 8 for 20 h, or were mock-infected before harvesting and staining with the indicated NCR-Fc fusion protein or the anti-HA mAb VVI-4G9 to monitor infection efficiencies. (C) For comparison, HeLa cells were infected using the same conditions and also stained with NKp30-Fc, NKp46-Fc or anti-HA.
(EPS)

Figure S2 **NKp30-Fc and NKp46-Fc bind to plate-coated viral particles and recombinant HA.** (A, B) Enzyme-linked immunoassays using 96-well plates coated with viral particles (VV strains WR and Lister, HA-deficient VV(WR)), with recombinant His_6_-tagged HA ectodomains from VV and ECTV, or with recombinant ICOS-L as an irrelevant protein. Coated and washed ELISA plates were reacted with NKp30-Fc, NKp46-Fc and NKp44-Fc, followed by incubation with goat anti-hIgG horseradish peroxidase(HRP)-conjugated secondary antibodies. The binding of viral particles was confirmed using the anti-HA mAb VVI-4G9 and goat anti-mIgG horseradish peroxidase(HRP)-conjugated secondary antibodies or polyclonal rabbit anti-VV for VV:ΔHA particles, respectively. In (A) and (B), some wells were incubated for 15 min with the anti-HA mAb VVI-4G9 before addition of NCR-Fc. In (B), NKp30-Fc was also preincubated with soluble recombinant HA(VV)-V5-His_6_ proteins before addition to the virus-coated ELISA plate (*„NKp30-Fc + sVV(HA*)*“*). The lack of binding of NKp30/NKp46-Fc to VV:ΔHA particles and the partial inhibitions observed in the presence of anti-HA or soluble HA underpin the HA-specific binding. (C) Western blot showing SDS-PAGE-separated, recombinant V5-His_6_-tagged HA(VV) and HA(ECTV) ectodomains, and V5-His_6_-tagged ICOS-L as a control protein, reacted with a mAb specific for the V5 epitope.
(EPS)

Figure S3 **Analysis of glycan-based NCR ligands on VV-infected and uninfected cells.** Uninfected (A) and VV-infected (B) HeLa cells were left untreated or treated with a mixture of heparanase I and III, or with hyaluronidase (*left column*), or deglycosylated with protein *N*-glycanase F for removal of *N*-glycans, a cocktail of *O*-deglycosylating enzymes, or with neuraminidase alone (*right column*), as described in Supplemental Materials and Methods (Text S1). After enzymatic treatment and washing, cells were immediately stained on ice with NKp30-Fc, NKp46-Fc or NKp44-Fc fusion proteins complexed with goat anti-hIgG-PE secondary antibodies. Heparanase I/III and hyaluronidase treatment reduced the staining of uninfected cells by the three NCR, but enhanced the staining of infected cells by NKp30-Fc and NKp46-Fc. Desialylation caused a strong enhancement of NKp44 labeling, whereas it had a reducing effect on NKp30-Fc binding to infected cells. (C) NP-40 lysates of VV-infected HeLa („H“) cells and murine NIH-3T3 („3“) cells, were either left untreated or digested with endoglycosidase H (Endo H), *N*-glycosidase F, neuraminidase or *O*-glycosidase, respectively. The lysates were separated by SDS-PAGE and a Western blot reacted with anti-HA mAb VVI-4G9 and peroxidase-conjugated anti-mIgG antibodies. The dominant, fully glycosylated, long form of HA (85 kDa) [42] and the Endo H-sensitive, intracellular short form of HA (68 kDa) are labeled with „L“ and „S“. The major HA form contained ∼5 *N*-glycans and ∼2 *O*-glycans as estimated from respective shifts in molecular weight after enzymatic deglycosylations. After neuraminidase treatment a minor portion of anti-HA-reactive molecules from HeLa lysates migrated at an apparently higher molecular weight (*asterisks*). These bands probably represent unseparated aggregates of HA.
(EPS)

Figure S4 **ECTV infection results in reduced NKG2D ligand expression.** HeLa cells infected for 20 h with VV (*black lines*) or ECTV (*gray lines*) were stained with mAb VVI-4G9 to monitor HA surface expression, with soluble NKG2D IgG-Fc fusion protein and with mAbs recognizing the NKG2D ligands MICA, MICB, ULBP1, ULBP2, ULBP3, or ULBP4, as indicated. Uninfected controls are shown with filled histograms. Infection with ECTV, but not VV, caused a downmodulation of the NKG2D ligands MICA, MICB and ULBP3.
(EPS)

Figure S5 **Cytofluorometric analysis of target and effector cells used in the cytotoxicity assay of** **Figure 4A** **.** (A) Infection time-dependent increase of HA surface and intracellular EGFP expression in HeLa cells infected for 6, 12 and 18 h, respectively, with wild-type VV(WR) or VV(WR):ΔHA[EGFP^+^] is shown in the top panel. The time-dependent increase of NKp30-Fc and NKp46-Fc binding to HeLa cells infected with wild-type VV for 12 and 18 h, but not to cells infected with VV:ΔHA, is shown below. (B) Stainings of primary NK cells from donor 1 (*black lines*) and donor 2 (*gray lines*) with PE-conjugated mAbs against NKG2D, NKp30, NKp46 or NKp44. The binding of a PE-labeled control antibody (MOPC-21) is presented as filled histogram.
(EPS)

Figure S6 **Inhibition of NK lysis by poxviral HA.** (A) Uninfected or VV-infected HeLa cells were ^51^Cr-labeled and used in a cytotoxicity assay with primary NK cells preincubated with either soluble recombinant HA-V5-His_6_ derived from VV(WR), or an irrelevant recombinant protein (ICOSL-V5-His_6_). Different E:T ratios are shown. (B) Lysis assay with primary NK cells as effectors and uninfected or VV-infected HeLa cells which were preincubated with either an irrelevant mAb (MOPC21) or with anti-HA mAb VVI-4G9. NK cells were preincubated with an excess of the blocking anti-CD16 mAb CLB-FcR gran/1 in order to prevent Fc receptor binding. (C) HeLa transfected either with the empty pcDNA3.1(+) vector for control, or with pcDNA3.1(+)/(HA)ECTV, were used in a lysis assay using NK-92 as effector cells. Where indicated, target cells were preincubated with an excess of soluble NKp30-Fc. Statistical comparison of samples with recombinant soluble HA-V5-His_6_ *versus* samples with control protein (A), samples with anti-HA *versus* samples with control mAb (B), and samples with NKp30-Fc *versus* without NKp30-Fc (C) were done using Student's *t*-test (paired, 2-tailed), *, p<0.05, **, p<0.01, ***, p<0.001.
(EPS)

Figure S7 **VV-infected stimulator cells reduce secretion of IFN-γ and TNF-α by primary NK cells.** Enzyme-linked immunoassays for human TNF-α (A) and IFN-**γ** (B) secreted into the culture medium of primary IL-2-activated NK cells cultivated together with uninfected HeLa cells, HeLa cells infected with wild-type VV(WR), or HA-deficient VV(WR), respectively, for 24 h. Prior to incubation with NK cells, the stimulator cells were treated with 254 nm UV light to prevent viral spread. For control, NK cells were also cultured alone. Statistical comparison of infected *versus* non-infected HeLa cells was done using Student's *t*-test (2-tailed), *n.s*., not significant, ***, p<0.001.
(EPS)

Text S1 **Supplemental materials and methods.**
(DOC)
