# Supplementary material for: Modulation of NKp30- and NKp46-Mediated Natural Killer Cell Responses by Poxviral Hemagglutinin
Source: PLoS Pathog. 2011 Aug 25;7(8):e1002195. doi: 10.1371/journal.ppat.1002195 (PMC3161980; doi:10.1371/journal.ppat.1002195)
Supplement: Text S1 — Supplemental materials and methods. (DOC) [file ppat.1002195.s008.doc]

**Supplemental Materials and Methods**

Cloning and Expression of B7-H6

Full-length B7-H6 cDNA (accession no. CR749521) linked to a GFP tag was cloned into the retroviral expression vector pMOWS+ containing a puromycin resistance gene. Retroviral transduction was done using the packaging cell line Phoenix-Ampho (American Type Culture Collection, Manassas, VA). Transduced Ba/F3 and HeLa were selected in puromycin and FACS-sorted for cells stably expressing B7-H6::GFP. Expression was verified by flow cytometry. Transfectants were cultured in DMEM containing 10% fetal calf serum, 1% penicillin/streptomycin and 1 μg/ml puromycin. For the culture of Ba/F3 cells, 0.1% 2-mercaptoethanol (2-ME) was included. PCR analysis of B7-H6 cDNA was performed on HeLa cell lysates using the primer pair 5’-GAG GAG GTC GAC ATG ACG TGG AGG GCT GCC GC-3’ and 5’-CTC CTC GCG GCC GCC CTG TAG GGG TAA CAG TAA AGT TGG GGG TTG-3’.

Antibodies

MAbs recognizing the NKG2D ligands MICA (M673), MICB (M360), ULBP1 (M295), ULBP2 (M310), ULBP3 (M550), and ULBP4 (M475) were kindly provided by D. Cosman (AMGEN, Seattle, WA). Anti-NKp46 (clone 195314), anti-NKp44 (clone 253415), anti-NKp30 (clone 210845), and the NKG2D-Fc chimeric receptor were purchased from R&D Systems (Bad Nauheim, Germany). Phycoerythrin(PE)-conjugated mAbs to NKp46 (9E2), NKp44 (2.29), and NKp30 (AF29-4D12) were from Miltenyi Biotec (Bergisch-Gladbach, Germany) and PE-labeled anti-NKG2D (1D11) was from BioLegend (San Diego, CA). The anti-CD16 CLB-FcR gran/1 (5D2) and a chicken polyclonal antibody against BAT3 (ab37751) were from abcam (Cambridge, UK). The irrelevant IgG1 mAb MOPC21 was from Beckman Coulter (Krefeld, Germany).

PE-conjugated goat anti-mouse Ig was from BD Biosciences/ Pharmingen (Heidelberg, Germany). HiLyte Fluor 647-labeled rabbit anti-chicken IgY was from AnaSpec (San Jose, CA). PE-conjugated goat anti-human IgG (Fc fragments) antibody and peroxidase-conjugated goat anti-human IgG/Fc, goat anti-mouse IgG/Fc as well as goat anti-rabbit IgG secondary antibodies were from Dianova (Hamburg, Germany).

Mouse monoclonal anti-B7-H6 antibodies were produced by immunizing mice with Ba/F3 cells transfected with B7-H6::GFP and boosting with purified B7-H6::ILZ fusion protein as described previously [71]. Antibodies were tested for specificity for B7-H6 by enzyme-linked immunosorbent assay, Western blot and flow cytometry analysis. Clone #16 was selected for further experiments. Hybridoma cells were cultured in RPMI-1640 medium supplemented with 10% fetal calf serum, 1% penicillin/streptomycin, 0.1% 2-ME, 0.1 mM sodium pyruvate and 25 mM HEPES.

Enzyme-linked immunosorbent assays (ELISA)

ELISAs with coated viruses were performed essentially as described [33]. Briefly, 96-well MicroTest III ELISA plates (BD Biosciences) were coated overnight at 4°C with ~10^7^ p.f.u. VV/well in 100 µl 0.05 M NaH_2_CO_3_-Na_2_HCO_3_ buffer (pH 9.6). Alternatively, ~1 µg soluble, recombinant V5-His_6_-tagged HA(VV), HA(ECTV), or ICOS-L as an irrelevant control protein, were coated. Plates were blocked using 5% skimmed milk powder (Merck) in PBS/0.05% Tween-20 (Sigma-Aldrich) (PBS-T) for 2 h at 4°C. NKp44-Fc, NKp46-Fc and NKp30-Fc (1 µg/100 µl in PBS-T with 1% BSA), anti-HA mAb 4G9 (~0.5 µg/well) or rabbit anti-VV antibodies (~1 µg/well), were added for 1 h at room temperature (r.t.) followed by 3 washes with PBS-T. For blocking of NCR-Fc binding, virus-coated wells were preincubated with anti-HA mAb 4G9 (~2 µg/well) for 15 min. Alternatively, soluble HA(VV)-V5-His_6_ (~1 µg) was preincubated with NKp30-Fc (1 µg/100 µl in PBS-T with 1% BSA) for 15 min before addition of the complex to virus-coated wells. Peroxidase-conjugated goat anti-human IgG/Fc, goat anti-mouse IgG/Fc or goat anti-rabbit IgG were added in PBS-T (1:3000 in PBS-T with 1% BSA) for 1 h at r.t. followed by 3 washes with PBS-T. Next, peroxidase substrate solution (Sigma-Aldrich; 1 mg/ml *o*-phenylenediamine in 0.1 M KH_2_PO_4_ buffer [pH 6.0], 0.03% H_2_O_2_), was added as for 20 min at r.t. in the dark. The substrate reaction was stopped with 50 µl 4 N H_2_SO_4_ and analyzed using a Titertek Multiscan Plus MKII ELISA photometer (MP Biomedicals, Heidelberg, Germany) at 492 nm.

IFN-γ and TNF-α release assays

Cytokine release assays were performed as described [33]. HeLa cells were left uninfected, infected with VV(WR) or VV(WR):ΔHA at m.o.i. 4 for 20 h. Subsequently, cells were harvested, washed and treated with short-wave UV light using a UV transilluminator (Bachofer, Reutlingen, Germany; 254 nm, 2 mW/cm^2^, ~10 cm distance) for 5 min. 10^5^ infected or uninfected cells were plated in triplicate 96-wells together with 10^5^ IL-2-preactivated primary NK cells for 24 h in complete RPMI medium. UV irradiation induced apoptosis in uninfected and infected stimulator cells. Supernatants were harvested, and quantification of TNF-α and IFN-γ was performed using a quantitative sandwich ELISA according to the manufacturer’s instructions (Human TNF-α and IFN-γ Quantikine kits; R&D Systems).

Enzymatic deglycosylation of cells and HA in lysates

For *N*-deglycosylation, 5x10^5^ uninfected or VV-infected HeLa cells were washed once in PBS and digested with 23 mU protein *N*-glycosidase F in 100 µl PBS/1% FCS [pH 7.4] for 1 h at 37°C. For *O*-deglycosylation, a mixture of enzymes (5 mU α2-3,6,8,9-neuraminidase, 3 mU β1,4-galactosidase, 1.3 mU endo-α-*N*-acetylgalactosaminidase, 40 mU β1-2,3,4,6-*N*-acetylglucosaminidase) (all from Merck/Calbiochem) were added in 100 µl PBS/1% FCS [pH 7.4] for 1 h at 37°C. For desialylation, α2-3,6,8,9-neuraminidase was used alone. Cells were incubated with hyaluronidase (type IV-S, Sigma-Aldrich) at 300 µg/ml in PBS or with a mixture of 100 mU heparinase I and 100 mU µl heparinase III (both from Sigma-Aldrich) in 100 µl PBS/1% FCS for 1 h at 37°C. After two washes in PBS/1% FCS, cells were labeled with NCR-Fc fusion proteins complexed with PE-conjugated goat anti-human IgG/Fc.

Hela cells were infected with VV using m.o.i. 4. 20 h after infection cells were harvested and lysed. Lysates were used for protein digestion with endoglycosidase H, *N*-glycosidase F, acylneuraminyl hydrolase and *O*-glycosidase (all from Roche Applied Science, Indianapolis, IN) according to the manufacturer’s instructions for 8 hours at 37°C. Digested lysates were separated by SDS-PAGE and transferred to nitrocellulose membranes. Immunoblot analysis was performed using anti-HA mAb VVI-4G9 and peroxidase-labeled goat anti-mouse IgG. Blots were developed by enhanced chemiluminescence.

References

71. Watzl C, Peterson M, Long EO (2000) Homogenous expression of killer cell immunoglobulin-like receptors (KIR) on polyclonal natural killer cells detected by a monoclonal antibody to KIR2D. Tissue Antigens 56: 240–247.
